# Clinical Significance of Elevated S100A8 Expression in Breast Cancer Patients

**DOI:** 10.3389/fonc.2018.00496

**Published:** 2018-11-05

**Authors:** Dujuan Wang, Guohong Liu, Balu Wu, Li Chen, Lihua Zeng, Yunbao Pan

**Affiliations:** ^1^Department of Clinical Pathology, Houjie Hospital of Dongguan, The Affiliated Houjie Hospital of Guangdong Medical University, Dongguan, China; ^2^Department of Radiology, Zhongnan Hospital of Wuhan University, Wuhan University, Wuhan, China; ^3^Department of Hematology, Zhongnan Hospital of Wuhan University, Wuhan University, Wuhan, China; ^4^Department of Laboratory Medicine, Zhongnan Hospital of Wuhan University, Wuhan University, Wuhan, China

**Keywords:** breast cancer, S100A8, relapse, estrogen receptor, immunohistochemistry

## Abstract

Breast cancer is the leading cause of female cancer-related death; however, novel biomarkers for predicting cancer recurrence still need to be explored. Aberrant expression of S100A8 has been reported to be related to tumor progression in various cancer types. This study aims to evaluate the clinical significance of S100A8 expression in breast cancer patients. In this study, data from 140 breast cancer patients were retrospectively collected to examine the association between S100A8 expression and clinical prognosis. Increased S100A8 expression was detected in breast cancer patients with relapse. The patients with increased S100A8 levels had significantly shorter disease-free survival (DFS) and overall survival (OS). In a multivariate survival analysis, a high histological grade and an elevated S100A8 level were independent factors associated with poor DFS and OS. Moreover, S100A8 expression was correlated with clinical subtype in breast cancer patients. The results showed that ER-negative and triple-negative breast cancer (TNBC) patients had significantly higher expression of S100A8 than patients with other subtypes. In conclusion, this study identified S100A8 as a potential biomarker for relapse in breast cancer patients.

## Introduction

Breast cancer remains the leading cause of cancer-related death in Chinese women, the average age of which is 10 years earlier than that in foreign countries (1). Systemic therapy, including hormonal treatment, targeted (trastuzumab) treatment, and cytotoxic chemotherapy, is the predominant treatment for breast cancer patients. The molecular bases of systemic therapy are expression patterns of estrogen receptor (ER), progesterone receptor (PR), and human epidermal growth factor receptor 2 (HER2) (2). Hormonal treatment is a good choice for ER- or PR-positive cases, and trastuzumab is a targeted medicine for cases with overexpression of HER2 (HR^−^/HER2^+^) (3). Triple-negative breast cancer (TNBC) patients lacking specific molecular markers are usually treated with cytotoxic chemotherapy. In general, the 5 years disease-free survival (DFS) rate is 89% in patients with well-treated, localized breast cancer; however, after a variable period of time, progression occurs because of recurrence (4, 5). It is necessary to explore a new and practical biomarker to predict relapse in breast cancer patients.

S100A8, which is a low molecular weight calcium-binding protein, is originally discovered as an immunogenic protein secreted by granulocytes and monocytes. S100A8 has emerged as an inflammatory factor and is associated with cancer. S100A8 is increasingly recognized as a biomarker in many solid tumors, such as head and neck squamous cell carcinoma (6), as well as colon (7), ovarian (8), bladder, and breast cancers (9). Recently, studies have shown that S100A8 is a potential indicator of poor survival in acute myeloid leukemia (AML) (10), and increased mRNA levels of S100A8 were associated with progression in breast cancer (11). S100A8 and S100A9 usually form a heterodimer called calprotectin, which is expressed in immune cells infiltrating the breast cancer stroma (12). S100A8/A9 stimulates breast cancer progression by activating key signaling pathways, such as IL-6-JAK2-STAT3, TNF-α-CXCL1/2, TGF-β, NF-κB, and MAPK (12, 13). A few studies have uncovered that individual S100A8 plays a vital role in poor prognosis of breast cancer. Infiltrating S100A8+ myeloid cells coordinated by macrophage inhibitory factor (MIF) result in poor overall survival (OS) and shorter metastasis-free survival in breast cancer patients (12). However, studies strongly suggest that S100A8 is expressed by cancer cells as well as by infiltrating immune and myeloid cells (14). The aim of this study was to examine the expression of S100A8 at the protein level in breast cancer cells.

We aimed to investigate the correlations between S100A8 and clinical parameters in 140 breast cancer patients and assess the clinical prognostic value of S100A8 expression in the relapse of breast cancer patients. We also elucidated the different expression levels of S100A8 in breast cancer patients with different subtypes.

## Materials and methods

### Patients and tissue samples

Breast cancer tissues from 140 patients were collected between March 2005 and December 2013 at Affiliated Houjie Hospital of Guangdong Medical University (Dongguan, Guangdong, PR China). We obtained all samples as formalin-fixed, paraffin-embedded (FFPE) tissue blocks for hematoxylin and eosin (HE) and immunochemical (IHC) analyses. The subtyping of the breast cancer samples was performed according to the clinical pathology guidelines for breast cancers. In total, 30 para-carcinoma tissue samples and 140 invasive breast cancer samples were assessed for their expression levels of S100A8, as shown in Table **2**.

### Ethics statement

This study was carried out in accordance with the recommendations of the human biomedical research guidelines from the Ethics Research Committee of Houjie Hospital. The protocol was approved by the Ethics Research Committee of Houjie Hospital. All subjects gave written informed consent in accordance with the Declaration of Helsinki.

### Treatment administration

All of the 140 samples from breast cancer patients were primary tumors. Patients were treated with surgery and adjuvant chemotherapy; 38 patients had a relapse, and 29 patients died from the cancer by September 2016. Cisplatin, paclitaxel, and anthracycline drugs were used in breast cancer chemotherapy.

### Hematoxylin and eosin (HE) staining and immunohistochemistry

The obtained breast cancer tissues were stained by HE following the usual pathology procedures. All of the cases were reviewed by two senior pathologists according to the WHO Classification of Tumors of the Breast (WHO2012) to determine the histological characteristics. A total of 140 FFPE human breast cancer specimens and 30 para-carcinoma samples were analyzed. The tissues were sectioned and dewaxed and then subjected to heat-mediated epitope retrieval using antigen retrieval reagent-basic (R & D Systems, #CTS013). After that, the tissues were incubated with an anti-human S100A8 monoclonal antibody (R & D Systems, MAB4570-SP) or cytokeratin 7 (CK7) antibody (Santa Cruz, sc-23876) overnight, incubated with an anti-mouse secondary antibody, and then subjected to the liquid DAB substrate-chromogen system. Specific staining of S100A8 protein was localized to the nucleus and cytoplasm of cancer cells. The expression level of S100A8 was evaluated in at least 200 tumor cells. All tests were performed according to the clinical pathological protocols of Affiliated Houjie Hospital of Guangdong Medical University.

### Tissue samples and western blotting

Specimens from patients who had undergone surgery for breast cancer were snap-frozen in liquid nitrogen and stored at −80°C. Paired samples of normal human breast epithelium and breast carcinoma were homogenized in RIPA lysis buffer. The lysates were resolved on 10% SDS-PAGE, transferred to PVDF membranes, and probed with primary polyclonal antibody to S100A8, using enhanced chemiluminescence reagents. β-actin (Sigma) served as internal positive controls.

### Detection of correlations between S100A8 expression and patient survival using clinical mRNA microarray analysis

An R2 microarray analysis and visualization platform (http://r2.amc.nl) was used as previously described (15) to determine whether the expression level of S100A8 was correlated with relapse status in breast cancer. We used the breast tumor dataset (Tumor Breast-Bergh-159-MAS5.0-u133a) and selected the analysis with “View a gene.” A similar method was applied to investigate the expression levels of S100A8 in different subtypes of breast cancer. The dataset of Tumor Breast-Wang-286-MAS5.0-ul133a was used to determine mRNA levels of S100A8 in breast cancer patients with different ER statuses. Then, we chose another breast tumor dataset (Tumor Breast-HER2-negative-Wessels-178) to investigate the differences in mRNA levels of S100A8 between the triple-negative group and the other groups.

Transcriptome data from patient samples of breast cancer were analyzed using the online database ONCOMINE (16) (https://www.oncomine.org/) to investigate whether S100A8 expression is associated with breast cancer. Prognostic Database PROGgeneV2 (17) (http://watson.compbio.iupui.edu/chirayu/proggene/database/index.php) was used to determine whether the expression level of S100A8 was correlated with DFS and the OS of breast cancer patients. We entered the S100A8 gene and plotted Kaplan-Meier curves with breast cancer patient data.

### Statistical analysis

The definition of breast cancer with relapse was regional or distant recurrence in any other position throughout the body. OS was defined as the period from initial diagnosis to death attributable to any cause. DFS was defined as the time from initial diagnosis to relapse, metastasis or death. The data between groups were analyzed with *t*-tests. The differences between low or high expression of S100A8 and clinical pathological variables were analyzed by a Chi-square test. We chose logistic regression analysis to identify the risk factors impacting relapse. The Kaplan-Meier method was used to plot the OS curves and DFS curves. Finally, we assessed the effect of multiple variables on survival curves by Cox regression analysis. The receiver operating characteristic (ROC) curves were generated to evaluate relapse and ER statuses in breast cancer patients. All the statistical analyses were performed using IBM SPSS 20 software (SPSS, Chicago, IL). All the results were considered statistically significant at *P* < 0.05.

## Results

### Patient characteristics and demographics

**Figure 1:**
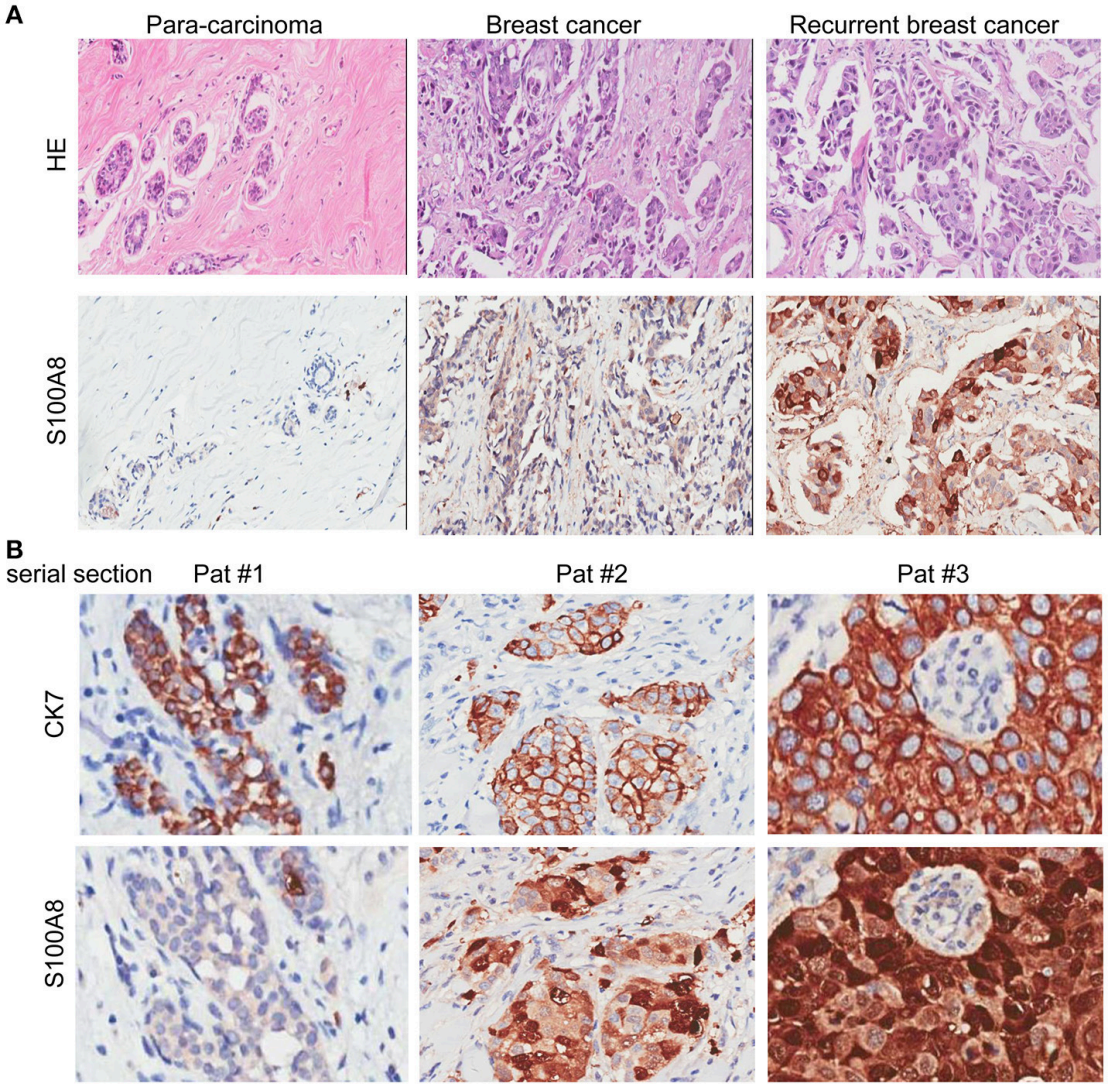
HE and IHC staining of para-carcinoma, invasive breast cancer, and recurrent breast cancer tissue samples. **(A)** HE staining (top) and immunohistochemical staining (bottom) of the three types of tissue: para-carcinoma (left), invasive breast cancer (middle), and recurrent breast cancer (right). **(B)** Immunohistochemical analysis of CK7 (top) and S100A8 (bottom) expression with serial section in three patients.

Samples from 140 patients with invasive breast cancer (median age, 54 years; range, 29–87 years) were used in the present study. We selected 30 cases with para-carcinoma tissue from the 140 samples at random to determine the expression of S100A8 at the protein level in a control group. The clinical characteristics, including age; tumor position; tumor size; and ER, PR, and HER2 statuses, are described in Table 1. The HE staining results of para-carcinoma, breast cancer and recurrent breast cancer tissues are shown in Figure 1A.

**Table 1:** Demographic characteristics of breast cancer patients assessed for S100A8 expression (*n* = 140).

**Variable**	**Total *n* = 140**	**Low-S100A8**	**High-S100A8**	***p***
**AGE**				
< 60	93	44 (47.3%)	49 (52.7%)	0.075
≥60	47	30 (63.8%)	17 (36.2%)
**POSITION**				
Left	71	36 (50.7%)	35 (49.3%)	0.616
Right	69	38 (55.1%)	31 (44.9%)
**HISTOLOGICAL TYPE**				
Ductal	130	66 (50.8%)	64 (49.2%)	0.103
Nonductal	10	8 (80%)	2 (20%)
**STAGE**				
I	33	18 (54.5%)	15 (45.5%)	< 0.001
II	61	42 (68.9%)	19 (31.1%)
III	46	14 (30.4%)	32 (69.6%)
**GRADE**				
G1	8	5 (62.5%)	3 (37.5%)	0.086
G2	101	58 (57.4%)	43 (42.6%)
G3	31	11 (35.5%)	20 (64.5%)
**TUMOR SIZE**				
T1	57	34 (59.6%)	23 (40.4%)	0.410
T2	81	39 (48.1%)	42 (51.9%)
T3	2	1 (50%)	1 (50%)
**LN METASTASIS**				
Yes	65	29 (44.6%)	36 (54.4%)	0.089
No	75	45 (60%)	30 (40%)
**ER STATUS**				
positive	79	53 (67.1%)	26 (32.9%)	< 0.001
negative	61	21 (34.4%)	40 (65.6%)
**PR STATUS**				
positive	60	43 (71.7%)	17 (28.3%)	< 0.001
negative	80	31 (38.8%)	49 (61.2%)
**HER2 STATUS**				
positive	29	9 (31.0%)	20 (69.0%)	0.012
negative	111	65 (58.6%)	46 (41.4%)
**RELAPSE**				
Yes	38	12 (34.2%)	26 (65.8%)	0.002
No	102	62 (60.8%)	40 (39.2%)
**MENOPAUSE**				
Yes	69	40 (58.0%)	29 (42.0%)	0.248
No	71	34 (47.9%)	37 (52.1%)

*LN, lymph node*.

### Association between S100A8 and clinicopathological parameters

The expression level of S100A8 in breast cancer cells was detected by IHC. Correlations between S100A8-positive cases and clinicopathological parameters are summarized in Table 1. The expression of S100A8 at the protein level was correlated with tumor stage (*p* < 0.001), ER status (*p* < 0.001), PR status (*p* < 0.001), HER2 status (*p* = 0.012), and relapse (*p* = 0.002) in breast cancer patients.

### Elevated S100A8 expression predicts relapse in breast cancer patients

IHC analysis was performed to detect the expression levels of S100A8 in para-carcinoma and breast cancer tissue from patients without and with relapse (Figure 1A). To exclude the influence from the tumor microenvironment, we also stained the serial section with both cytokeratin 7 (CK7) and S100A8. As expected, the CK7 positive cells (tumor cells) were also positive for S100A8 (Figure 1B).

**Figure 2:**
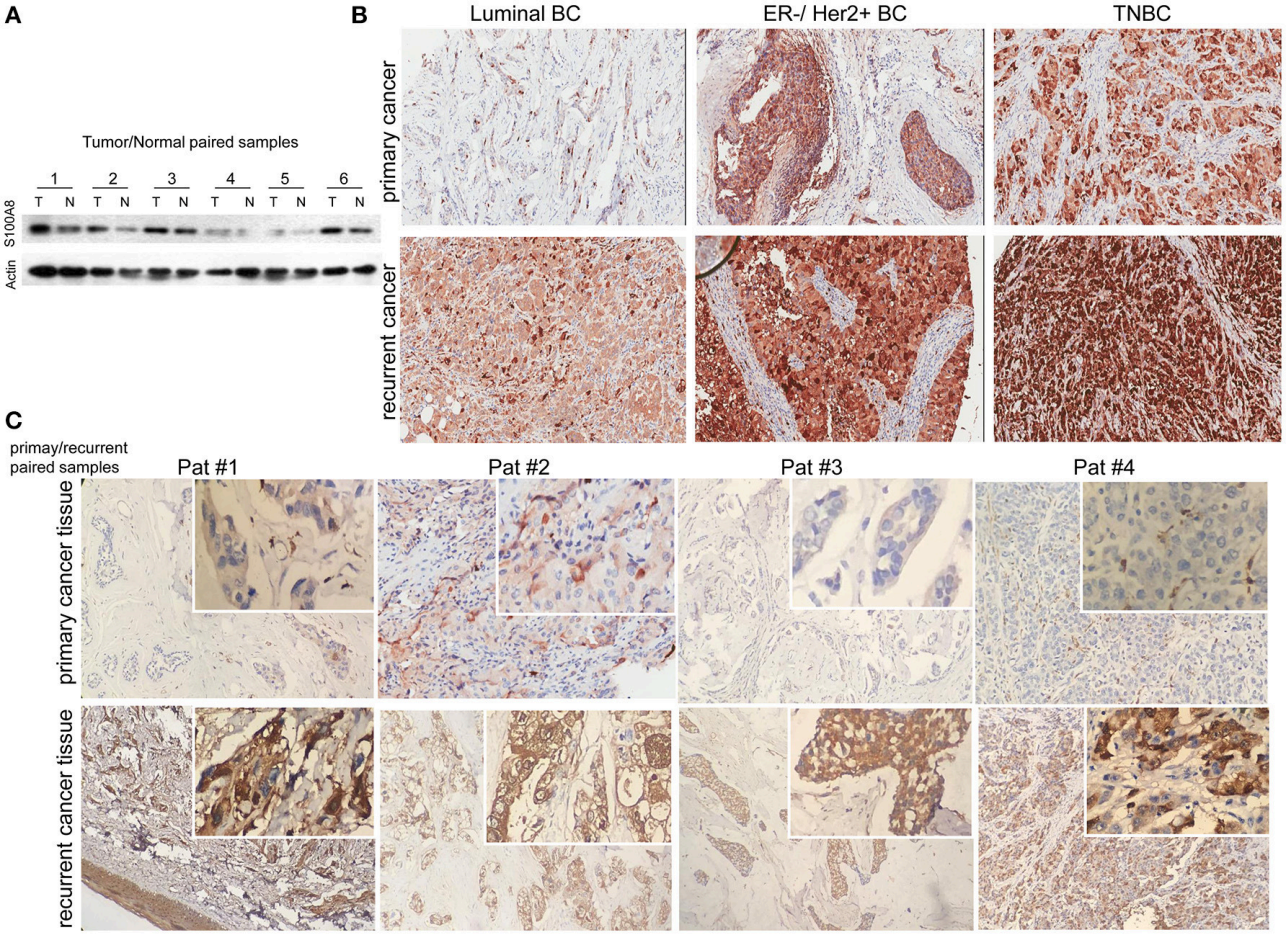
Immunohistochemical analysis of S100A8 expression in different subtypes of breast cancer tissue samples. **(A)** Western blots of paired samples of tumor tissue (T) and non-neoplastic breast tissue (N) immunoblotted against S100A8 or β-actin (used as a loading control). **(B)** IHC staining for S100A8 expression in primary (top) and recurrent (bottom) breast cancer tissue samples: luminal breast cancer (left), ER-negative and HER2-positive breast cancer (middle), and TNBC (right). **(C)** IHC analysis of S100A8 expression in primary/recurrent paired samples.

To explore the significance of S100A8 in human breast cancer progression, we further examined whether S100A8 protein expression was different in paired normal human breast epithelium and breast carcinoma samples by immunoblotting methods. Figure 2A shows a dramatically increased S100A8 level in four of six tumors as compared with the paired normal tissue, which show little S100A8 expression.

**Figure 3:**
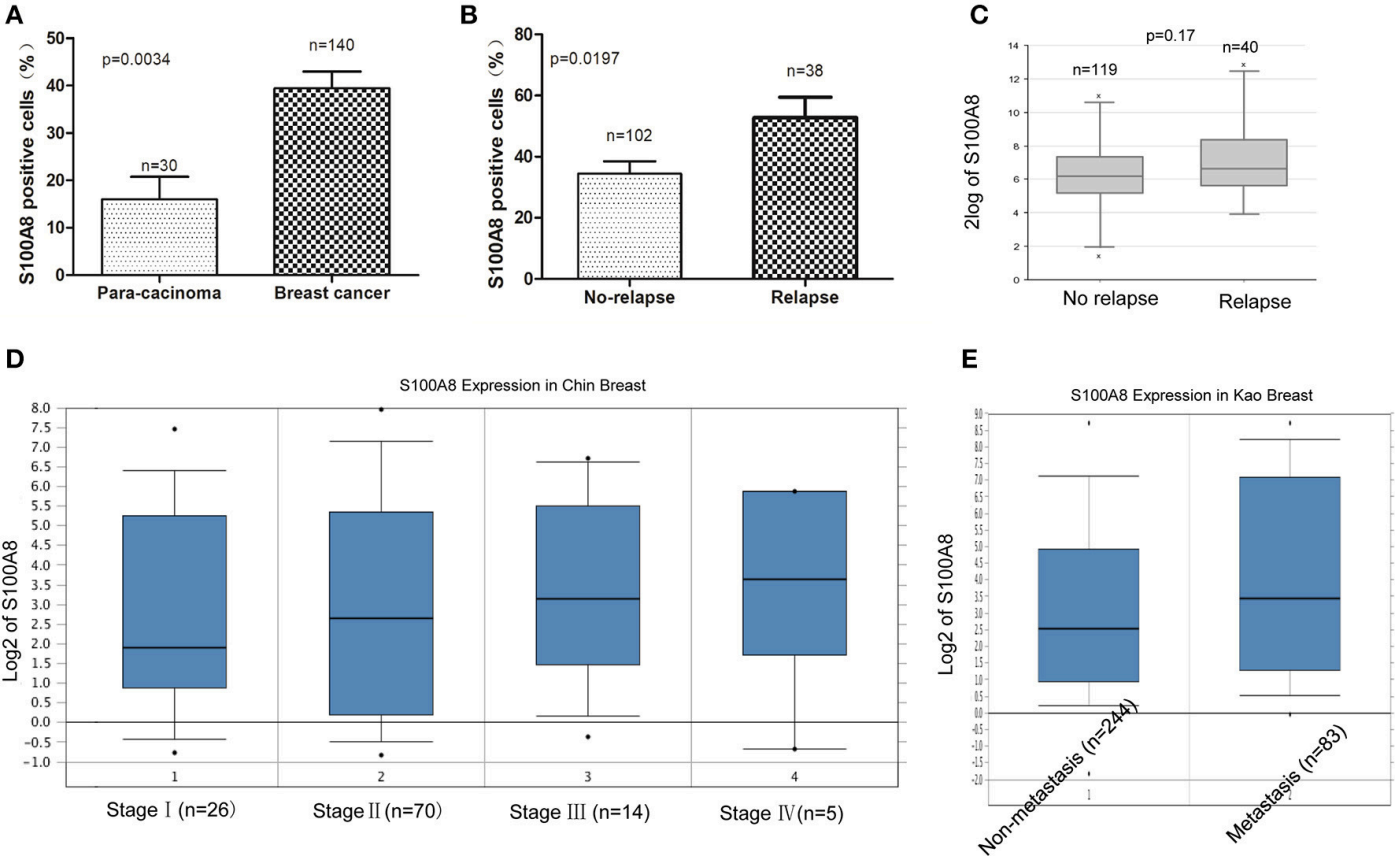
S100A8 is overexpressed in breast cancer tissue. The percentages of S100A8-positive cells in para-carcinoma and breast cancer tissue **(A)** and in breast cancer tissue without relapse or with relapse **(B)**. S100A8 gene expression in breast cancer patients without relapse or with relapse was plotted using the R2 online database **(C)**. S100A8 gene expression in breast cancer patients with different stage **(D)** or patients without metastasis or with metastasis **(E)** was plotted using Oncomine database.

It was demonstrated that the percentage of S100A8-positive cells in recurrent breast cancer patients was significantly higher than that in breast cancer patients without recurrence (Figure 2B, Figure 3). We further investigated the S100A8 expression in four cases of primary and recurrent paired samples, and found that S100A8 expression were significantly elevated in recurrent samples than that in paired primary samples (Figure 2C). The analysis from the online database showed that the average expression of S100A8 at the mRNA level in recurrent breast cancer patients was also higher than that in breast cancer patients without relapse, but there was no significant difference between the two groups (Figure 3C). Furthermore, data from the online database Oncomine demonstrated that S100A8 expression increased as the tumor stage increased (Figure 3D). The average expression of S100A8 in metastatic breast cancer patients was also higher than that in breast cancer patients without metastasis (Figure 3E).

We used logistic regression analysis to identify risk factors impacting recurrence in breast cancer patients. We found that increased S100A8 expression at the protein level and PR status were associated with relapse (*p* < 0.05, Table 2).

**Table 2:** Logistic regression analysis of factors predicting recurrence in breast cancer patients (*n* = 140).

**Parameters**	**B**	**Wald**	**Sig**.	**Exp (B)**	**95% CI**
Age (≥60 y)	−0.333	0.214	0.643	0.717	0.175–2.937
Position	0.075	0.026	0.872	1.078	0.435–2.671
Histological type	0.965	0.579	0.447	2.624	0.219–31.510
Grade	−0.550	0.222	0.637	0.577	0.059–5.666
Stage	1.094	1.245	0.265	2.985	0.437–20.385
Tumor size	−1.093	2.803	0.094	0.335	0.093–1.205
LN metastasis (+)	0.644	1.103	0.294	1.905	0.572–6.339
ER (+)	−0.788	1.695	0.198	0.455	0.137–1.508
PR (+)	1.270	4.044	0.044	3.560	1.033–12.275
HER2 (+)	0.560	1.018	0.313	1.751	0.590–5.198
Menopause (+)	0.494	0.542	0.462	1.693	0.440–6.104
S100A8 (high)	1.297	5.593	0.018	0.359	1.249–10.723

*LN, lymph node*.

### Expression patterns of S100A8 in breast cancer patients with different subtypes

**Figure 4:**
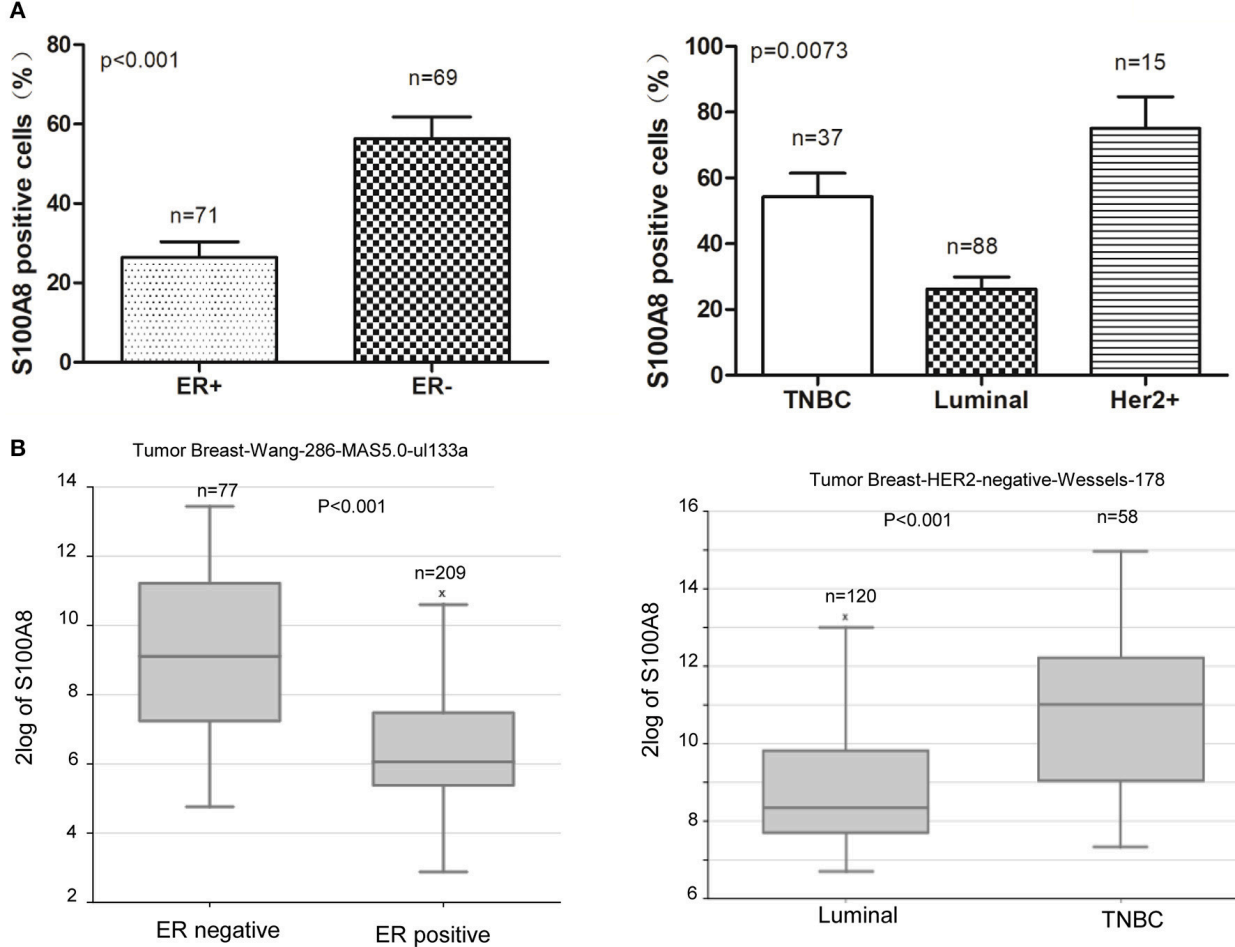
S100A8 is overexpressed in ER-negative breast cancer tissue. **(A)** The percentages of S100A8-positive cells in different ER status breast cancer tissue samples (left) and triple-negative (TNBC) and luminal breast cancer tissue samples (right). **(B)** S100A8 gene expression in breast cancer patients with different subtypes of breast cancer was plotted using the R2 online database.

In our study, we found that the percentage of S100A8-positive cells was higher in ER-negative breast cancer patients than that in ER-positive patients. The expression level of S100A8 was higher in TNBC subtype patients than that in luminal subtype patients (Figures 2, 4). It was suggested that increased S100A8 expression at the protein level was associated with ER status in breast cancer patients (Figure 4A), which was in agreement with the analysis from the online database. The results showed that the expression of S100A8 at the mRNA level in TNBC and ER-negative breast cancer patients was higher than that in luminal subtype breast cancer patients (Figure 4).

### Association of S100A8 expression with cancer patients

**Figure 5:**
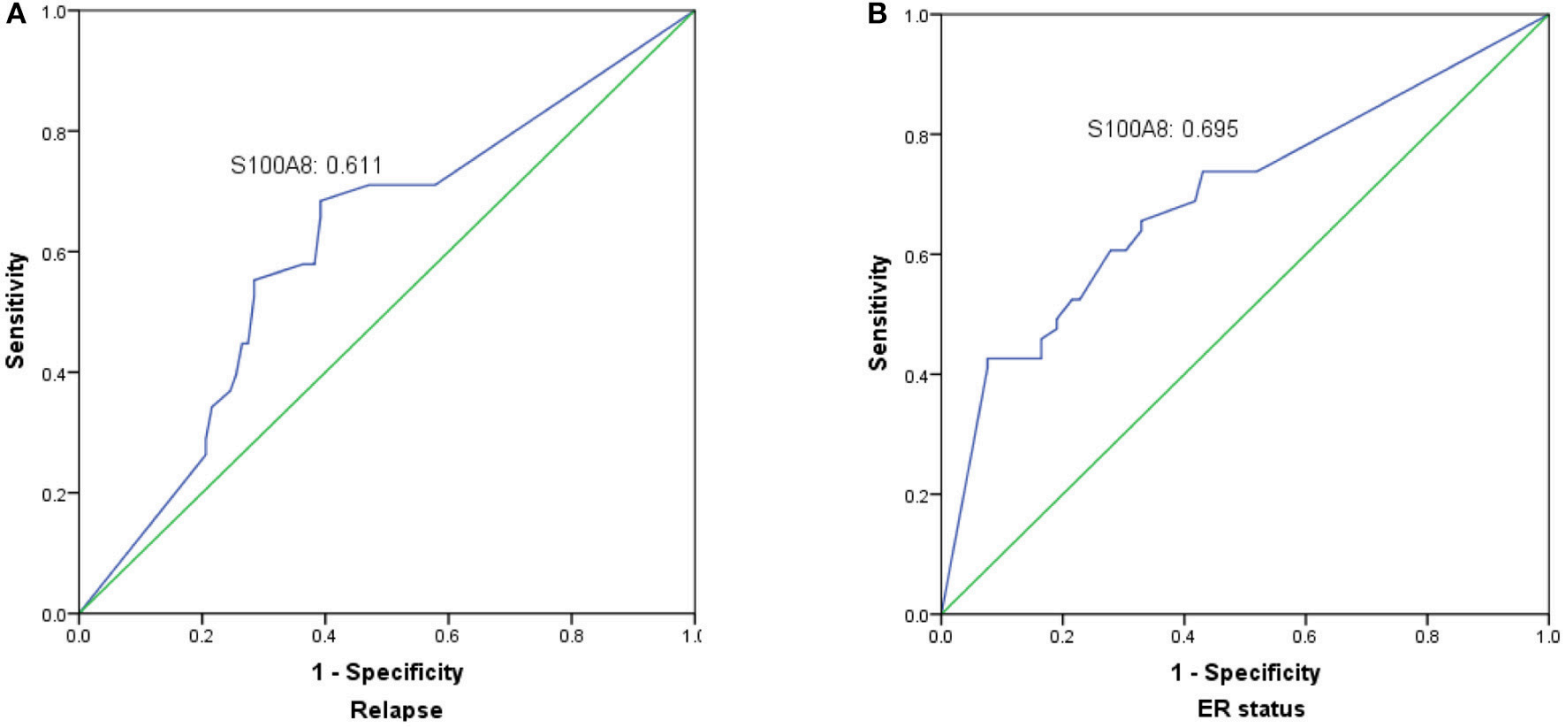
The significance of S100A8 expression was analyzed by establishing an ROC curve in breast cancer. The curve illuminated that elevated S100A8 expression could indicate the possibility of relapse **(A)** and ER status **(B)** in breast cancer patients.

The prognostic value of S100A8 was investigated in breast cancer by ROC curve analysis. The AUC value of the ROC curve was 0.611 for S100A8 (Figure 5A), with a sensitivity of 54.1% and a specificity of 70.9%. We also analyzed the relationship between S100A8 and ER status. The AUC value of the ROC curve was 0.695, while the sensitivity was 42.6%, and the specificity was 92.4% (Figure 5B).

### Survival analysis

**Figure 6:**
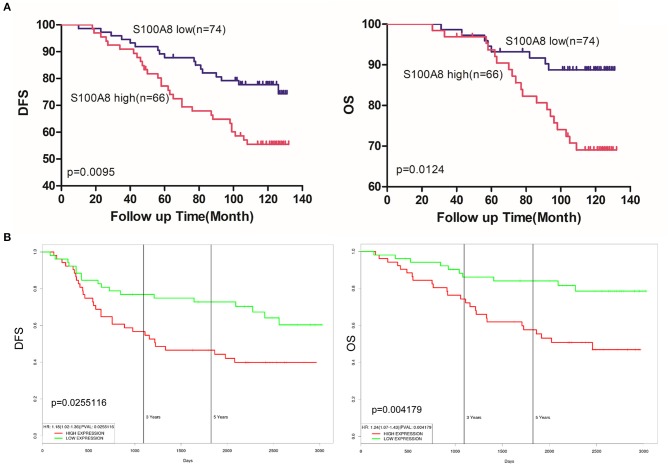
S100A8 expression predicts survival in breast cancer. **(A)** Kaplan-Meier analysis of the correlations between S100A8 expression and DFS and OS in breast cancer patients. **(B)** Kaplan-Meier plots of the online breast cancer database (Prognostic Database PROGgeneV2) were used to assess associations between S100A8 gene expression and patient survival in breast cancer.

Among the 140 patients with breast cancer, 29 died before September 2016. The mean follow-up time of patients was 108 months [range 26–132 (OS) and 10–132 (DFS)]. Kaplan-Meier analyses were used to compare the S100A8 low and high expression groups of breast cancer patients. The survival curves suggested that patients with high levels of S100A8 had a significant association with worse DFS (*p* = 0.0095) and OS (*p* = 0.0124) (Figure 6A). We also evaluated the association between the elevated expression of S100A8 and overall or relapse-free survival in breast cancer patients in online databases and showed similar results (Figure 6B). These data suggested that a high expression level of S100A8 was associated with worse survival in breast cancer.

### Prognostic factors

We used COX analysis to evaluate the effects of multiple variables, including age, tumor location, histological type, tumor grade, tumor stage, tumor size, lymph node metastasis, ER status, PR status, HER2 status, menopause status and S100A8 expression level, on OS and DFS. The results showed that tumor grade (OS: *p* = 0.021; DFS: *p* = 0.030) and S100A8 expression (OS: *p* = 0.003; DFS: *p* = 0.002) were significant prognostic factors for breast cancer patients (Table 3). Multivariate analysis confirmed that increased S100A8 expression was an independent factor for poor outcome in breast cancer patients.

**Table 3:** Multivariate survival analysis for OS and DFS in breast cancer.

**Parameters**	**OS**		**DFS**	
	***P***	**HR (95% CI)**	***P***	**HR (95% CI)**
Age (≥60 y)	0.108	2.801 (0.798–9.833)	0.299	1.702 (0.624–4.639)
Position	0.395	1.415 (0.636–3.148)	0.364	1.339 (0.713–2.512)
Histological type	0.945	1.087 (0.102–11.584)	0.955	0.955 (0.197–4.641)
Grade	0.021	1.477 (0.256–8.511)	0.030	6.682 (1.206–37.012)
Stage	0.537	2.160 (0.187–24.891)	0.080	5.152 (0.821–32.313)
Tumor size	0.906	0.936 (0.311–2.811)	0.151	0.544 (0.237–1.249)
LN metastasis	0.638	1.489 (0.284–7.811)	0.991	0.993 (0.267–3.689)
ER	0.057	0.373 (0.135–1.030)	0.240	0.622 (0.282–1.373)
PR	0.202	0.520 (0.190–1.420)	0.822	1.097 (0.491–2.449)
HER2	0.476	1.419 (0.542–3.719)	0.154	1.743 (0.811–3.743)
Menopause	0.446	1.595 (0.480–5.303)	0.569	1.320 (0.508–3.428)
S100A8 (high)	0.003	2.434 (1.349–4.393)	0.002	2.094 (1.308–3.352)

*DFS, disease-free survival; HR, hazard ratio; CI, confidence interval; and LN, lymph node*.

## Discussion

Although numerous studies have worked to clarify the mechanisms of prognosis and drug resistance in breast cancer, reliable and valuable biomarkers for the prediction of relapse are limited. S100A8 is dysregulated in many kinds of cancers. However, the prognostic role of S100A8 is still elusive. The prognostic role of S100A8 has mainly been demonstrated in hematological malignancies (10, 18). However, few studies have explored the prognostic role of S100A8 as a biomarker in solid tumors.

S100A8-positive cells detected in breast cancer stroma are usually expressed by infiltrating immune and myeloid cells. Currently, few studies have reported that S100A8 is also expressed by breast cancer cells (9). Choi J et al. detected S100A8 by IHC staining of invasive ductal carcinoma (IDC) and metastatic carcinoma in the lymph nodes (MSN). The research showed that the expression of S100A8 was associated with a higher stage (19). In our study, we found similar results. However, studies evaluating the prognostic role of S100A8 protein expression in cancer cells are still rare.

In our study, we measured the expression of S100A8 in breast cancer tissue by immunohistochemical staining. Our results revealed that S100A8 was expressed in cancer cells and that the average number of S100A8-positive cancer cells in tissues from patients with relapse was much higher than that in tissues from patients without relapse. Our study revealed that S100A8 was a promising predictor for relapse in breast cancer patients. S100A8 was highly expressed in patients with shorter OS and shorter DFS. The Cox regression analysis demonstrated that elevated S100A8 expression and tumor grade were independent prognostic factors for poor survival in breast cancer patients. Therefore, the detection of S100A8 expression by IHC might have prognostic value in breast cancer.

S100A8 is coded at the chromosome locus 1q21.3, and Goh et al. have reported that amplification at 1q21.3 is present in more than 70% of recurrent breast cancers (13). Most studies of S100A8 have revealed that the S100A8 mRNA level is increased in breast cancer tissue from patients with poor prognosis (20). However, Bao et al. reported that there was no association between high mRNA expression of S100A8 and poor OS (21). In our study, we confirmed that the mRNA level of S100A8 was increased in relapsed breast cancer patients, and an elevated expression level of S100A8 was significantly correlated with shorter OS and DFS in public microarray datasets.

Furthermore, in our study, we uncovered the different protein expression levels of S100A8 in different clinical subtypes. We revealed that the ER status of breast cancer patients had a close association with S100A8 protein expression. The expression of S100A8 in ER-positive breast cancer patients was obviously lower than that in ER-negative patients. Miller et al. demonstrated that increased S100A8 protein expression was an independent prognostic indicator of poor outcome in ER-positive tumors (9). Drews-Elger et al. demonstrated that the infiltration of S100A8-positive myeloid cells was associated with basal-like breast cancer, resulting in poorer outcomes in breast cancer patients (12). In our study, we first discovered that S100A8 expression was significantly higher in TNBC patients than that in luminal patients. Therefore, the mechanism controlling different expression levels of S100A8 in subtypes of breast cancer remains to be discovered.

In conclusion, increased S100A8 protein expression was associated with poor survival and a higher probability of relapse in breast cancer patients. Furthermore, we demonstrated elevated S100A8 expression in ER-negative and TNBC patients. These data indicated that S100A8 could be a potential biomarker for breast cancer.

## Author contributions

DW, GL, and BW performed the experiments. LC and LZ reviewed the medical records and collected data. YP and DW conceived the study, analyzed the data, and wrote the manuscript.

### Conflict of interest statement

The authors declare that the research was conducted in the absence of any commercial or financial relationships that could be construed as a potential conflict of interest.

## Abbreviations

ER: estrogen
TNBC: triple negative breast cancer
BC: breast cancer
HER2: human epidermal growth factor receptor 2
OS: overall survival
DFS: disease-free survival.
